# A Strategic Approach to Use Upcycled Si Nanomaterials for Stable Operation of Lithium-Ion Batteries

**DOI:** 10.3390/nano11123248

**Published:** 2021-11-30

**Authors:** Junghwan Kim, Jisoo Kwon, Min Ji Kim, Min Ju O, Dae Soo Jung, Kwang Chul Roh, Jihyun Jang, Patrick Joohyun Kim, Junghyun Choi

**Affiliations:** 1 Energy Storage Materials Center, Korea Institute of Ceramic Engineering and Technology, Jinju 52851, Korea; wndel6@naver.com (J.K.); wltn0704@naver.com (J.K.); 191008@kicet.re.kr (M.J.K.); dhalswn1031@kicet.re.kr (M.J.O.); dsjung@kicet.re.kr (D.S.J.); rkc@kicet.re.kr (K.C.R.); 2 Department of Applied Chemistry, Kyungpook National University, Daegu 41566, Korea; 3 Department of Chemical and Biological Engineering, Seoul National University, Seoul 08826, Korea; cjh7228@hanmail.net

**Keywords:** silicon anode, nanostructured Si, upcycled Si, state of charge control, lithium ion battery

## Abstract

Silicon, as a promising next-generation anode material, has drawn special attention from industries due to its high theoretical capacity (around 3600 mAh g^−1^) in comparison with conventional electrodes, e.g., graphite. However, the fast capacity fading resulted by a large volume change hinders the pragmatic use of Si anodes for lithium ion batteries. In this work, we propose an efficient strategy to improve the cyclability of upcycled Si nanomaterials through a simple battery operation protocol. When the utilization degree of Si electrodes was decreased, the electrode deformation was significantly alleviated. This directly led to an excellent electrochemical performance over 100 cycles. In addition, the average charge (delithation) voltage was shifted to a lower voltage, when the utilization degree of electrodes was controlled. These results demonstrated that our strategic approach would be an effective way to enhance the electrochemical performance of Si anodes and improve the cost-effectiveness of scaling-up the decent nanostructured Si material.

## 1. Introduction

In order to reduce greenhouse gas emission from the combustion of fossil fuels, the efficient use of electric energy without carbon emission has become more important than ever [1,2]. The rechargeable lithium-ion battery (LIB) is one of the most promising energy storage systems to support the net-zero carbon emissions. LIBs have a decent energy density and an excellent cycle durability, in comparison with other energy storage devices. However, the limited theoretical specific capacity of conventional electrode materials, e.g., graphite and LiCoO_2_, is the biggest hurdle before achieving the high energy density of LIBs [3]. During the last few years, the adoption of silicon (Si) as an anode has been in the spotlight due to its relatively low working potential (~0.4 V vs. Li/Li^+^) and high theoretical capacity (3600 mAh g^−1^) at the lithiation phase of Li_15_Si_4_ [4,5,6]. Unfortunately, the capacity fading originated from the significant volume change of Si electrodes during cycling discourages the use of pure Si electrodes [7].

To improve the electrochemical stability of Si electrodes, material research, such as the use of nano-sized Si, Si/carbon composite and surface-modified Si, has been extensively conducted [8,9,10,11,12]. Among these options, the nano-sized Si is considered the most attractive candidate. The serious electrode fracturing could be circumvented even during a repetitive lithiation/delithiation process, if the Si is below a certain critical size (150 nm) [9,13]. The surface modification of Si powder with a conductive material can further enhance the electrochemical performance in terms of cycle stability and rate capability [8,12]. Although the previous strategies of optimizing Si electrodes have shown a significant improvement in electrochemical properties, there are still remaining challenges with regard to scalability and cost-effectiveness before moving onto industrial applications [14].

Recycling/upcycling industrial wastes is a promising strategy to improve the scalability as well as address the inherent problems of bulk Si electrodes. Among many of the industrial wastes, Si sawdust derived from the photovoltaic (PV) industry would be a strong anode candidate, since approximately 40% of the Si ingot turns into waste after sawing [15,16]. A number of researchers have attempted to use the recycled Si for anode electrodes. However, further material processes such as composite-manufacturing, carbon-coating and NH_4_F treatment are additionally required to achieve decent electrochemical performances [14,16,17]. Considering this point, a simpler and more effective methodology should be proposed to improve the cost-effectiveness of material preparation.

In this work, we demonstrate an efficient approach to improve the electrochemical performances of upcycled Si nanomaterials as well as to reduce the material preparation cost. Nanostructured Si dust (NSD) was used as an active material without any further treatment. NSD has a similar capacity compared to bulk Si, but is capable of delivering a much better cycle retention than bulk Si. In addition, NSD has a strong advantage in reducing the manufacturing cost. The utilization degree (i.e., state of charge) of Si was controlled by changing the lower cut-off voltage. The Si anode with a different utilization degree showed a reduced volume change and improved cycle performances. The specific capacity of anodes reached more than 1500 mAh/g, which is a higher value than that of conventional Si materials, such as Si/C and SiO_x_ [12,18]. In addition, it was confirmed that the average charge potential of the anode was decreased under the controlled condition. It leads to a higher nominal voltage of full cells and, thus, an improvement in energy density.

## 2. Results and Discussion

**Figure 1:**
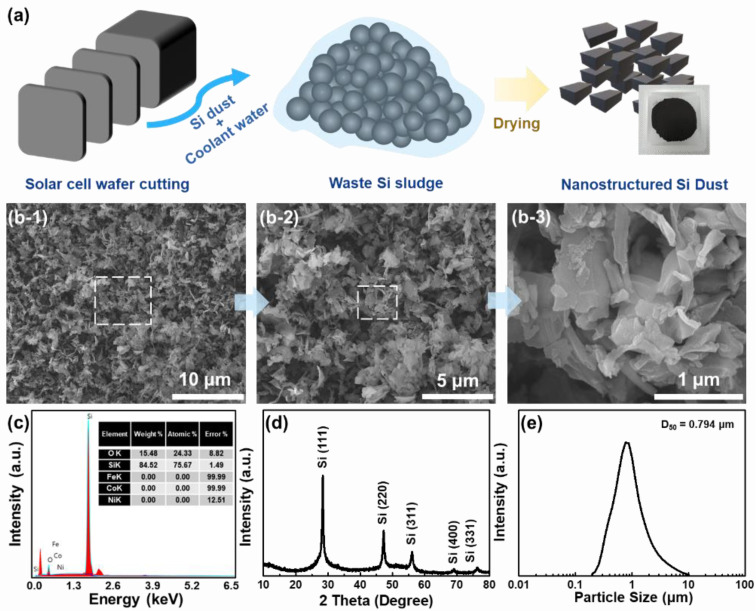
(**a**) Schematic illustration of yielding process of NSD, (**b1**–**b3**) scanning electron microscopy (SEM) images of NSD, (**c**) energy dispersive X-ray spectroscopy (EDX) results of NSD, (**d**) X-ray diffraction (XRD) pattern and (**e**) particle size distribution (PSD) of NSD.

Figure 1a shows a schematic illustration of the yielding process of NSD produced as a by-product from the manufacturing process of the Si wafer for a solar cell. The Si sludge with coolant solution is formed during the sawing process of the Si ingot, to make the Si wafer. After drying process under inert conditions, NSD powder can be obtained. As shown in Figure 1(b1–b3), NSD has a block-like morphology with a particle size below 1 μm. The distribution of the particle size of NSD was measured by particle-size analysis (Figure S1). The size of Si dust is ranging from 200 nm to 10 μm.

Since the majority of NSD is composed of nano Si, it is capable of maintaining the electrode structure without serious pulverization after multiple cycles. It is likely that NSD with block-like structure could enhance the cycle performance, since the nanostructured Si is able to alleviate stress formed during lithiation [11]. Most of the synthetic methods for designing the 2D-nanostructured Si have limitations on mass production, due to tricky and expensive procedures. In this regard, the preparation approach of upcycling Si sludge into nanostructured Si has a number of advantages in terms of commercialization.

Energy-dispersive X-ray spectroscopy (EDX) and X-ray diffraction (XRD) analysis were employed to confirm the presence of impurities in the NSD. There was no noticeable impurity peak in the EDX result (Figure 1c). The XRD result (Figure 1c) also supports that NSD has the typical crystalline structure of Si (JCPDS No. 27-1402), without impurity phases [19].

**Figure 2:**
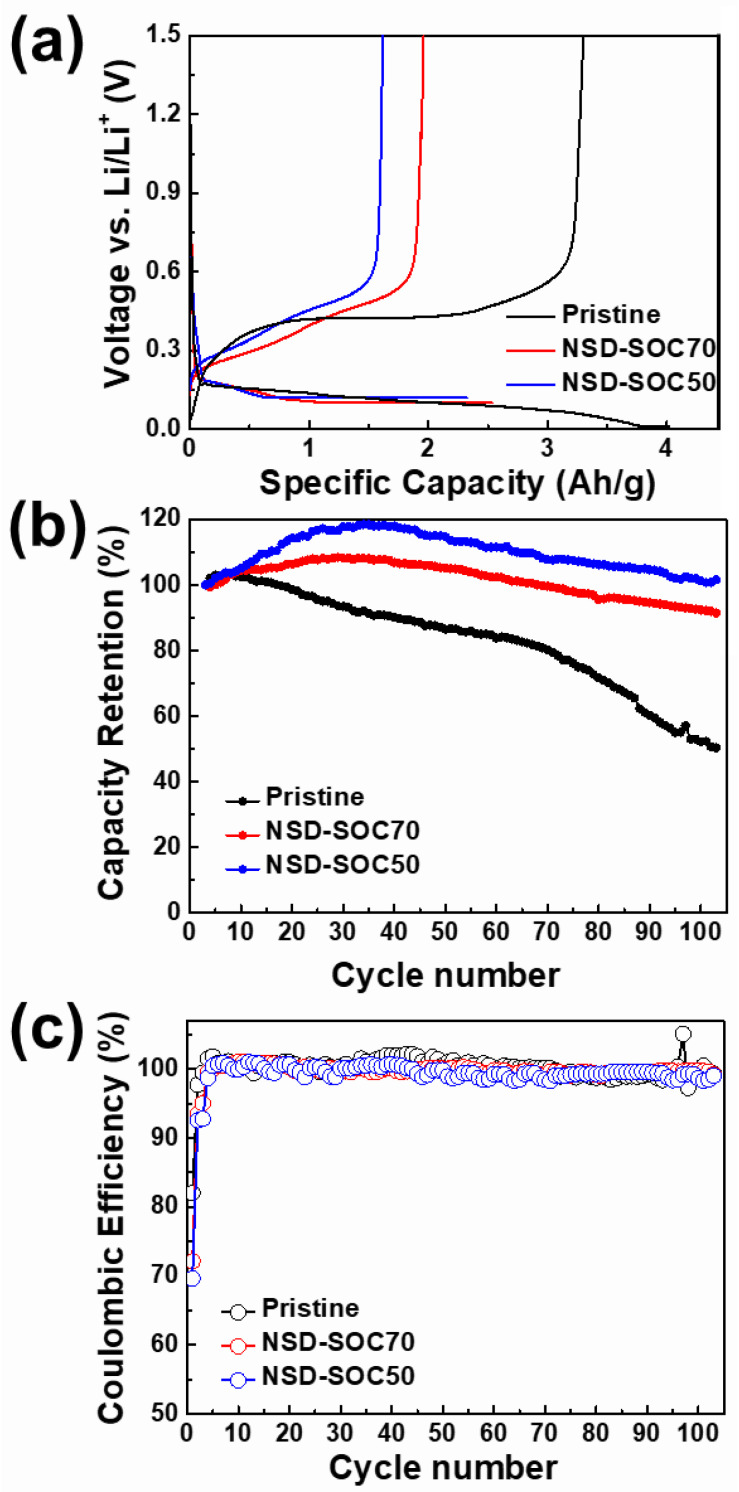
Electrochemical performances of NSD electrodes with different utilization degrees. (**a**) Initial cycle voltage profiles at 0.1 C, (**b**) cycle retention at 0.5 C and (**c**) Coulombic efficiency at 0.5 C.

Although the unique nanostructure and size distribution of NSD is beneficial to mitigate the stress generated during cycling, severe volume change still remains due to the large amount of Li^+^ incorporation into the Si. In order to investigate the influence of the lithium uptake degree on electrochemical performances, the state of charge (SOC) was controlled by changing the lower cut-off voltage. The cut-off voltage of pristine NSD (100% SOC), NSD-SOC70 (70% SOC) and NSD-SOC50 (50% SOC) were 0.01 V, 0.10 V and 0.12 V, respectively. The electrochemical properties of NSD electrodes were examined using 2032 coin-type half-cells. NSD-anode electrodes served as working electrodes and pure lithium metal foil was used as reference/counter electrodes. The initial discharge–charge curves of each electrode at 0.1 C are presented in Figure 2a. All electrodes exhibited two voltage plateaus at near ~0.1 and ~0.4 V in the lithiation and de-lithiation curves, which corresponds to the alloy reaction of the crystalline Si with lithium [8]. Additionally, there are no noticeable plateaus associated with side reaction, except for the SEI formation or impurity plateaus. Pristine NSD, NSD-SOC70 and NSD-SOC50 electrodes exhibited initial charge (de-lithiation) capacities of 3312 mAh/g, 1960 mAh/g and 1617 mAh/g, respectively. The cycle test was conducted at a current rate of 0.5 C (1 C = 2000 mAh/g) for 100 cycles (Figure 2b). The formation process (at 0.1 C for 1 cycle and at 0.2 C for 1cycle) was conducted to electrochemically activate the anode electrode and form a stable SEI layer before the cycling test. The capacities of pristine NSD, NSD-SOC70 and NSD-SOC50 electrodes retained 50.3%, 91.5% and 101.7% of initial charge capacity after 100 cycles, respectively. The capacity retention of NSD-SOC50 electrode showed more than 100% of its initial charge capacity due to the partial activation of the anode electrode. The partial activation of the electrode is capable of enhancing the cycle performance of the practical cell, since inactivated Si replaces dead Si during cycling. The high-capacity retention of SOC-controlled electrodes (NSD-SOC70 and NSD-SOC50) is attributed to the efficient prevention of material fracture and electrode pulverization arising from the volumetric change. Mechanically stable electrodes are beneficial to keep maintaining the structural integrity of a solid electrolyte interphase (SEI) layer during cycling, which is one of the most important aspects to design Si anodes [20].

**Figure 3:**
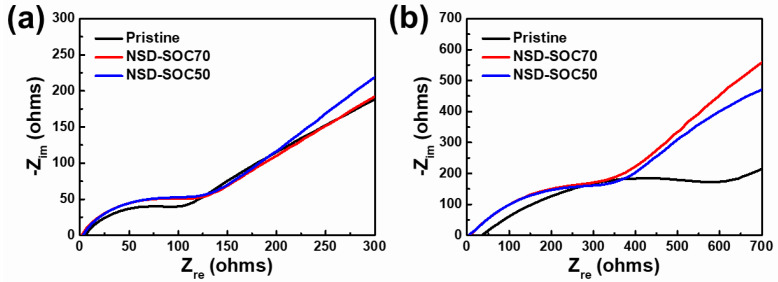
Electrochemical impedance spectra (EIS) of NSD electrodes before (**a**) and after cycling (**b**).

To compare the electrochemical performances between electrodes, electrochemical impedance spectroscopy (EIS) tests were carried out after 1 and 100 cycles in the frequency range from 250 kHz to 10 mHz with an amplitude of 5 mV. Nyquist plots obtained from EIS are plotted in Figure 3. The x-intercept of a semicircle indicates an Ohmic resistance and the diameter of the semicircle corresponds to a charge transfer resistance (R_ct_) [21,22]. The initial charge transfer resistances of pristine NSD, NSD-SOC70 and NSD-SOC50 electrodes are 95.5 Ω, 98.9 Ω and 117.4 Ω, respectively. The highest charge transfer resistance of NSD-SOC50 is related to the partial activation of anode. The changing ratio of charge transfer resistance of each electrode before and after cycling is significantly different. After 100 cycles, the pristine NSD has the highest charge transfer resistance (365.1 Ω) in comparison with NSD-SOC70 (190.1 Ω) and NSD-SOC50 (183.7 Ω). The huge increase in resistance indicates the degradation of the pristine NSD electrode (i.e., material fracture, electrode crack, unstable SEI, electrolyte depletion), which is attributed by the volume change of the Si electrode. Considering this result, the step to modulate the utilization degree of electrodes facilitates the stable cycling of electrodes as well as stabilizes the SEI layer during cycling.

**Figure 4:**
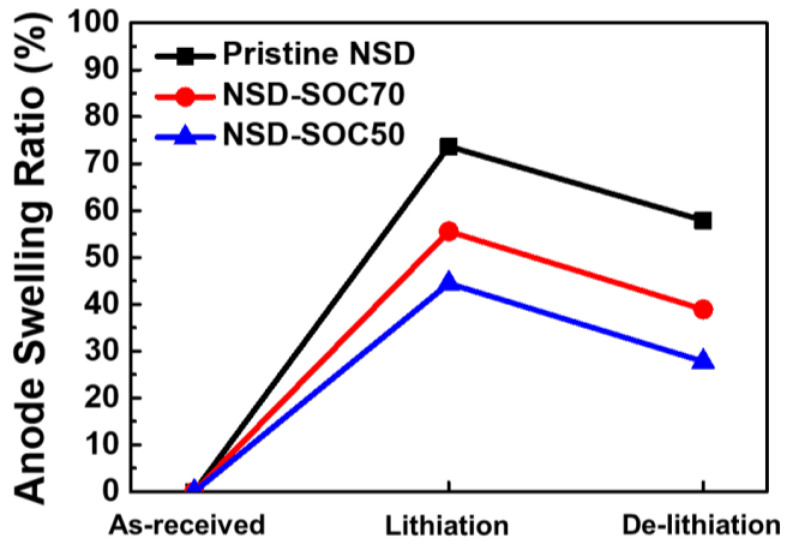
Swelling behavior of NSD electrodes.

In order to investigate the swelling behavior in terms of utilization degree, we measured the thickness change of each electrode after lithiation (Figure 4). After lithiation, the NSD electrode exhibited 73.7% of electrode swelling when compared to the as-received electrode. On the other hand, NSD-SOC70 and NSD-SOC50 showed a reduced swelling ratio of 55.5% and 44.4%, respectively. Transmission electron microscopy (TEM) results also corroborated that the electrode with controlled utilization degree maintained its structural integrity, even after a cycle. These results can be inferred from the morphology of electrode, i.e., material crack (Figure S1). In general, the reduced expansion of electrodes can minimize the pulverization of electrode components and provide an improved cycle stability. As shown in Figure 4, each electrode was still expanded even after the full delithiation. Pristine NSD, NSD-SOC70 and NSD-SOC50 had electrode-swelling ratios of 57.9%, 38.9% and 27.8%, respectively. This is because the delithiated Si electrode could not recover its original configuration due to the irreversible structural transformation. However, an interesting thing to note is that electrodes with controlled utilization degree are relatively more beneficial to maintaining the electrode structure than the fully charged NSD. It is reported that the structure distortion of electrodes accelerates a fading capacity. Moreover, the electrode swelling could raise safety issues caused by separator deformation or battery package breakage [23,24]. The enhanced structure resilience of electrodes with a controlled utilization degree can address the concerns with regard to capacity fading and safety problem.

**Figure 5:**
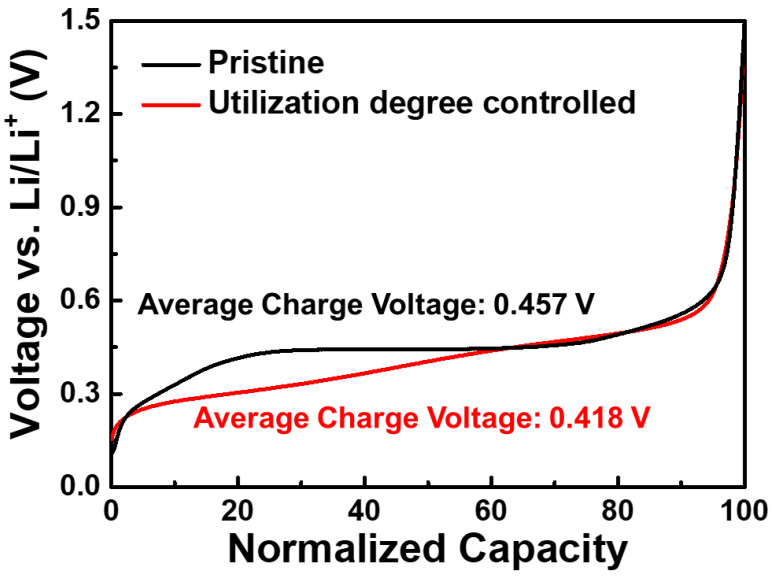
Normalized charge voltage profiles of a pristine NSD electrode and a controlled NSD electrode with different utilization degrees (NSD-SOC50).

The nominal voltage of LIBs is an important factor to determine energy density. However, the operating voltage of the Si anode is an intrinsic property, which cannot be changed by material engineering. Unlike the previous approach, controlling the utilization degree would be the only way to enhance the nominal voltage because the desired voltage plateaus range can be easily optimized according to the requirements. Figure 5 compares the voltage profiles of a pristine NSD electrode and controlled NSD electrodes with different utilization degrees (NSD-SOC50). NSD-SOC50 shows a lower average charge voltage (0.418 V) than pristine NSD (0.457 V), which leads to a higher nominal discharge voltage in full cells. This would contribute to an improvement in energy density.

In summary, we proposed an efficient strategy in order to improve the overall electrochemical performances and to practically use the upcycled Si nanomaterial (NSD) as an anode. The proposed battery operation protocol, i.e., control of utilization degree, was applied to the NSD electrodes. An NSD electrode has a similar specific capacity and superior cycle performance when compared to the previously reported bulk Si material. It is because the volume change of Si materials is significantly mitigated, leading to an improved electrical contact between the Si electrode and current collector. Apart from the electrochemical performance, our approach has a strong benefit in terms of fabrication cost, since the nanostructured Si material can be easily obtained and scaled up from the industrial waste. Considering these beneficial features, our proposed strategy would provide a key for designing Si materials suitable for a next-generation lithium ion battery and advance the development of practical Si anodes in a near future.

## 3. Experimental Section

### 3.1. Preparation of Nanostructured Si Dust Anode Electrode

Nanostructured Si dust was provided by TRS Co. Ltd. and used as an anode material without further treatment. The slurry was prepared by mixing 80% Si, 10% Super P and 10% polyacrylic acid (M_v_ ~450,000, Sigma-Aldrich, St. Louis, MO, USA), as well as deionized (DI) water, with a planetary mixer. This as-prepared slurry was laminated onto a copper foil via tape-casting. The casted electrode was dried in a vacuum oven at 100 °C for 3 h. The mass loading level of the Si electrode was 1.6 mg/cm^2^.

### 3.2. Characterization

The morphology of NSD was analyzed using a field emission scanning electron microscopy (FESEM, MIRA II LMH, TESCAN, Brno-Kohoutovice, Czech Republic). The chemical composition was characterized using energy-dispersive X-ray spectroscopy (EDX, Oxford Instruments, Abingdon, UK, attached to the FESEM). The crystallographic structure of NSD was investigated using an X-ray diffractometer (XRD, D8 Advance, Bruker, Billerica, MA, USA). Particle size analysis (PSA, LA-950V2, Horiba, Kyoto, Japan) was used to measure the particle-size distribution of NSD.

### 3.3. Measurement of Electrochemical Performance

Coin-type half-cells (2032R type) were fabricated to evaluate the electrochemical performances of anode electrodes. Lithium metal foil served as both the reference and counter electrode. Polyolefin membrane and 1.15 M lithium hexafluorophosphate (LiPF_6_) in ethylene carbonate-ethylmethyl carbonate-dimethyl carbonate (EC-EMC-DMC, 2:4:4 vol%), containing 12.5 wt% of FEC, were used as separator and electrolyte. The amount of electrolyte was 100 μL. The electrochemical properties were characterized under the galvanostatic mode at 0.5 C. Pre-cycling was conducted to form a stable SEI layer for three cycles (one cycle at 0.1 C, one cycle at 0.2 C and one cycle at 0.5 C). Electrochemical impedance spectroscopy (EIS) was evaluated in the frequency range from 250 kHz to 10 mHz at amplitude of 5 mV.

## Data Availability

The data presented in this study are available on request from the corresponding author. The data are not publicly available.

## Supplementary Materials

The following are available online at https://www.mdpi.com/article/10.3390/nano11123248/s1, Figure S1: Transmission electron microscope (TEM) images of utilization degree controlled electrode for before and after cycling.
