# Supplementary material for: A Strategic Approach to Use Upcycled Si Nanomaterials for Stable Operation of Lithium-Ion Batteries
Source: Nanomaterials (Basel). 2021 Nov 30;11(12):3248. doi: 10.3390/nano11123248 (PMC8703682; doi:10.3390/nano11123248)
Supplement: Supplementary file 1 [file nanomaterials-11-03248-s001.zip › nanomaterials-1475418-supplementary.pdf]

Supporting Information

A Strategic Approach to Use Upcycled Si Nanomaterials for Stable Operation of Lithium-Ion Batteries

Junghwan Kim [1,2,†], Jisoo Kwon [1,†], Min Ji Kim [1], Min Ju O [1], Dae Soo Jung [1], Kwang Chul Roh [1], Jihyun Jang [3], Patrick Joohyun Kim [2,*] and Junghyun Choi [1,*]

1 Energy Storage Materials Center, Korea Institute of Ceramic Engineering and Technology, Jinju 52851, Korea; wndel6@naver.com (J.K.); wltn0704@naver.com (J.K.); 191008@kicet.re.kr (M.J.K.); dhalswn1031@kicet.re.kr (M.J.O.); dsjung@kicet.re.kr (D.S.J.); rkc@kicet.re.kr (K.C.R.)
2 Department of Applied Chemistry, Kyungpook National University, Daegu 41566, Korea
3 Department of Chemical and Biological Engineering, Seoul National University, Seoul 08826, Korea; cjh7228@hanmail.net
* Correspondence: jchoi@kicet.re.kr (J.C.); pjkim@knu.ac.kr (P.J.K.)
† These authors contributed equally to this work.

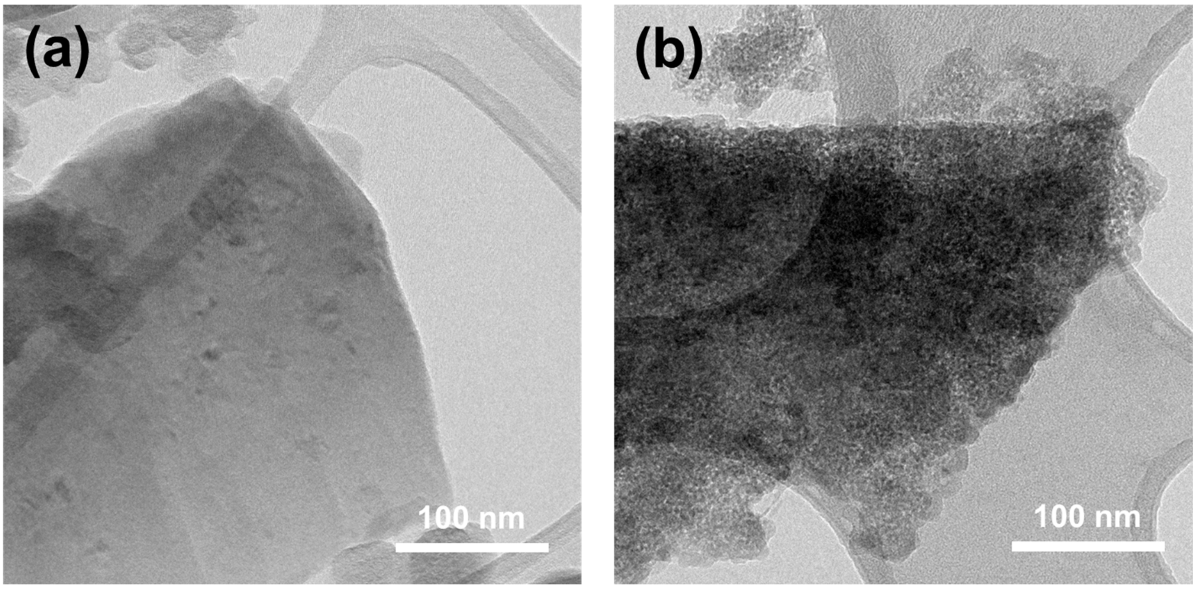

Figure S1 Transmission electron microscope (TEM) images of utilization degree controlled electrode for (a) before and (b) after cycling
